# Supplementary material for: Off-chip prefetching based on Hidden Markov Model for non-volatile memory architectures
Source: PLoS One. 2021 Sep 14;16(9):e0257047. doi: 10.1371/journal.pone.0257047 (PMC8439492; doi:10.1371/journal.pone.0257047)
Supplement: Attachment — Submitted filename: Response_To_Reviewers.pdf [file pone.0257047.s001.pdf]

Rebuttal Letter – July 2021
Paper:

Off-chip Prefetching Based on Hidden Markov Model for Non-Volatile Memory Architectures

On date June 9, 2021 notification from editor was received requesting a revised version of the manuscript. The authors consider the comments from editor and reviewers are justified and useful to improve our work so, consequently, we have made new experiments and deep changes in several sections in the manuscript. In this document we address all these changes.

Before explaining the changes made in response to the reviewers' comments, there are two important points that are worth highlighting:

- We have re-written the “Evaluation” section. We have made new experiments to get results from a 16-core processor. We have also developed a new model to assess how OS-managed multiprogramming influences our prefetcher's ability to manage complexity. We also have improved the figures and the explanation and justification of the results. The results now clearly show how our proposal behaves in isolation and in comparison to similar systems.

- Reviewer number 2 insists on several comments about the need to reference the equations in the section “Hidden Markov Model for VA Clustering”. We have re-read this section and improved some of the explanations because it is true that in some points it was difficult to understand. But the main point is that the mathematical narration is based on three basic principles (Bayes theorem, hidden markov model and least squared method) that are well known and developed many years ago, so they are not part of the bibliography. In short, what we have done is a mathematical development in which each equation arises from the previous one and from one of the three principles that I have already mentioned, so it is not possible to include a bibliographic reference to these equations because they have been generated by us.

Next, we answer in detail all the comments.

Editor Comments

Discussion about appropriate prefetch models for NVM and justification why Markov-based prefetch is the best option.

We have included a discussion in section “Prefetch Based on Hidden Markov Model” in which we present the different types of hardware prefetching with an updated bibliographic reference of the current proposals. We explain the lack of ability of these techniques to keep track of very complex miss data patterns with simultaneously identification of their corresponding group. This is very important in NVM architectures since they must efficiently manage big data and other complex applications. We also have included state-of-the-art NVM prefetch models in the “Related Work” section.

We consider that the Hidden Markov Model is very well suited to this problem because it fits exactly to one of the three canonical HMM problems: to find the set of transition states starting from an output sequence. In this case the output sequence is the LLC miss lines and the transition states is the group to which each line belongs. Once the groups are identified it is way easier to predict next LLC misses. We believe this is a very natural way to deal with the hardware prefetch problems in high complexity environments like an off-chip cache in a NV-RAM memory architecture. We have included this reasoning in the corresponding section.

Relevance of LLC in the evaluation of HMM performance and its connection with different NVM-RAM scenarios.

- A paragraph has been added in the introduction discussing one of the main scenarios or NVM-RAM organization, Fabric Attached Memory Architectures (FAM-NVM). We explain how this organization is fully compatible with our approach because it suppposes the existence of a small SDRAM memory attached to each compute node and FAM-NVM attached to network fabric. This is equivalent to our off-chip SDRAM cache so the problems to solve in both proposals are the same. The main problem is latency and our HMM prefetch proposal fits very well in this kind of memory architecture. We believe that this work increases the relevance of our proposal and shows how latency is one of the main problems to solve to have NVM architectures fully in use.
- In section "Related Work" we introduce similar current state-of-the-art works. Specifically [Kommareddy20] (PreFAM), [Choi21] and [Talati21] implement prefetch in the last level of the on-chip memory hierarchy, that is, the LLC. They show the relevance of prefetch techniques for external memory modules (SDRAM cache, small SDRAM memory modules) because of the high latency of NVM architectures.
- Also in section "Prefetch Based on Hidden Markov Model" we explain the on-chip memory hierarchy based on virtual address highlighting its advantages and disadvantages. Here we show that LLC analysis that relies on virtual addresses instead of physical addresses reduces drastically access frequency while providing enough information for a good prefetcher to catch algorithmic behavior and produce good prefetch accuracy. We've also added a comparation between the literature proposals and our HMM model highlighting our main differential proposal.

Reviewer #1 Comments

Latest approaches can be included in the related work

- In related work we have included three recent works in hardware prefetching related to different features of LLC, big data and machine learning, irregular workloads and fabric memory integration:
  - [Choi21]: LLC prefetcher targeted to big data and machine learning processing that adapt granularities entailing cache lines and page granularity. Includes a DRAM buffer with an access history table and several prefetch engines to deal with irregular memory access patterns.
  - [Talati21]: hardware-software codesign solution to deal with very irregular workloads that uses static information from programs and dynamic from hardware to extract program semantic information used to generate prefetch requests. Our proposal succeeds in capturing irregular data access patterns without the use of compiler information from running programs.
  - [Kommareddy20]: decoupling of memory from computing is currently under research because improves data bandwidth when used with novel interconnection technologies. This kind of memory organization eases the task of integration between different

technologies. The problem is the latency of memory access so the use of prefetch techniques has a very high impact in performance.

- Also a more updated bibliography is included related to the use of hardware prefetching in modern processors. This is added in the introduction. We have included a work of hardware prefetching in the Intel Xeon Phi ([Sodari16]) and other in the IBM BlueGene/Q ([Haring12]).
- Analysis or similar proposals like Domino [Bakhshlipour18] compared with our proposal is also included to explain how HMM Prefetch deals with more complex data relations on each of the groups it can simultenously identify (section “Prefetch Based on Hidden Markov Model”).

Provide proof for the performance of HMM when complexity increases.

In the section “Evaluation” we have improved the explanations and also we have created an specific section for multicore evaluation including new experiments with sixteen cores. We have also developed a new model to assess how OS-managed multiprogramming influences our prefetcher's ability to manage complexity. This model has a 4 core processor running 4 benchmarks each so multiprogrammed is needed to context switch between processes. With this new simulation model the complexity that time sharing adds to the off-chip access pattern can be evaluated. These modifications have been included to provide proofs ot the behavior of HMM Prefetch on more real and complex environments. We believe that now the evidence of how HMM works in very complex environments is sufficient.

More explanation required for HMM in prefetching

We have reviewed all the mathematical development and modified the writing to make the reasoning clearer. We believe now it is easier to understand and we would like to highligh that all our mathematical frame in the paper is based on three well known principles (Bayes theorem, hidden markov model and least squared method) and all we do is operate using them.

Reviewer #2 Comments

The writing is confusing and redundant. Bibliography is old and low quality.

All the text has been revised and improved. Abstract has been rewritten. Introduction has been completed with new NVM architectures to put in context our proposal. We have changed the captions in all figures trying to make them clearer. Evaluation section has been deeply revised and updated.

We have updated the bibliography to include current works related to our target problem, that is, high latency in off-chip memory access related to NVM architectures. At this moment, out of 37 references 23 (62%) are less than 5 year old and 14 (38%) are older. Some of the older references are necessary because they refer to very important topics or algorithms (Joseph97, Somogyi09, Young09, Ward63) others are necessary because are the basic reference of tools and benchmarks used in the work (Luk05 for pin tool and SpecCPU2006) so we must include them and, finally, others refer to survey techniques on commercial processors (Conway10, Haring12) and is difficult to find a more current version for this kind of information.

Related to the quality of the referenced work, it is very important to explain that in computer architecture is customary to use a small set of international conferences to publish the last research work. These conferences include ISCA, MICRO, HPCA and ASPLOS among others. For this reason some conferences have very high quality, at the same level than top journals. Out of the 37 references, 21 (56%) belong to this category and the remaining are necessary because they focus on very similar issues to those covered in this work. We also include a good number of top journal references. We do not believe that as a hole, our references can be classified as low quality work.

Figures are not very legible, lack of homogeneity and are poorly described in the text.

It is true that some of the figures weren't homogeneous and we hope now they are clearer and easier to understand. We have made these changes:

- Fig. 1. This figure is explained in the text but we also have improved the caption explanation. We've also improved its shape and legibility.
- Fig. 2, Fig. 3, Fig. 4. Previous explanation was difficult to understand so we have improved caption and text explanation.
- Fig. 5. We have improved its shape and legibility and, also, the caption explanations.
- Fig. 6. We improved its appearance since we consider it is well explained
- Fig. 7. We changed the legends and caption description. Also we have improved text description.
- Fig. 8 and Fig. 9. Changed the legends to improve legibility.
- Fig. 10. This figure has been changed modifying text and including colors in order to make it clearer. Text and caption have also been reviewed and changed to improve readability.
- Fig. 11, Fig. 12. We have improved the legends in the figure, the caption text and their description.

Equations presented are not justified and no reference source specified. Improve description of mathematical expressions.

We have re-read this section and improved some of the explanations because it is true that in some points it was difficult to understand. But the main point is that the mathematical narration is based on three basic principles (Bayes theorem, hidden markov model and least squared method) that are well known and developed many years ago, so they are not part of the bibliography. In short, what we have done is a mathematical development in which each equation arises from the previous one and from one of the three principles that I have already mentioned, so it is not possible to include a bibliographic reference to these equations because they have been generated by us. Of course it is possible to improve its description and we have tried to do it.

Restructure the document

We believe the document is well structured. A lot of work and thinking as been spent on it. Reviewer #1 and #3 agree with us. We also believe that the section "Evaluation" can be improved so we have reestructured this section including two new subsections, one of them dedicated to the analysis of more complex multicore systems and the other to the analysis of the complexity added

by the OS due to context switch of the processes. We believe now there is enough evidence of the behavior of our proposal.

The abstract is very extensive and has quite room for improvement

We have rewriten the abstract to clearly explain the problem that motivates our work, our proposal, its main characteristics, how it works and its ability to identify complexity and, finally, its improvement compared to similar proposals.

The use of the language is misused

We have made a great effort to review and improve the writing in all sections. Repetitive portions in the text have been removed and much others have been restructured and rewritten. We hope now the text is clearer and easier to understood than before.

The results framework must be improved. Contrast the results with respect to another modeling technique / performance index.

We have rewritten the *Evaluation* section improving and making clearer the explanations, modifying entire paragraphs and changing the order of others. A new set of experiments with a more complex multicore architecture has been done to compare the results with more complex computer systems. A new class of architecture has been evaluated to verify how the different prefetchers behave on a multiprogrammed multicore system. The performance index we use are standard (hit rate and overhead) allowing the comparative study with other systems.

Introduction: restructure to highlight novelty with more current information. Describe the real problem as a consequence of the bibliographic research.

We have included three more similar recent works (years 2020 and 2021) in the "Related work" section. These works show how the latency is today an important problem in NVM architectures due to the mix of the very irregular data access patterns and the high inherent latency of NVM modules. Different hardware prefetching techniques appear in current literature showing the insterest of this topic in the research community.

Related work: improve the contrast with current works to highlight novelty of our work

We have included current researcho work covering the same topic. We compare the characteristics and limitations of the diferent works against our proposal showing how we deal with some of those limitations by allowing a more complex and complete prefetch solution.

The "Related work" section should be described in the introduction

This section is described in the "Introduction".

Reviewer #3 Comments

Impact of HMM in the production final price.

The impact of our prefetch technique in the final production price is an important issue. Sometimes as researchers we are too focused in the design and technical topics and we forget that contributions to the state-of-the-art should also be applied in order to be useful to society. We appreciate the reviewer #3 comments and we have included, in subsection "Prefetcher Implementation", an study of the cost of an HMM implementation on an actual processor.
